# Neuroprotective Effects of *Senecio brasiliensis* Extract Against Chlorpyrifos‐Induced Neurotoxicity and Oxidative Stress in *Drosophila melanogaster*

**DOI:** 10.1111/bcpt.70288

**Published:** 2026-08-23

**Authors:** Maria Vitória Takemura Mariano, Giany Gabriely Padão dos Santos, Ana Beatriz Guedes de Souza, Thaís de Camargo Neves, Franklin Vinny Medina Nunes, Pedro Henrique Vicenzi, Karen Kich Gomes, Mauro Eugênio Medina Nunes, Marcelo Farina, Jeferson Luis Franco, Thaís Posser

**Affiliations:** ^1^ Oxidative Stress and Cell Signaling Research Group, Interdisciplinary Center for Biotechnology Research – CIPBIOTEC Federal University of Pampa São Gabriel Brazil; ^2^ Department of Biochemistry and Graduate Program in Biochemistry, Biological Sciences Center Federal University of Santa Catarina Florianópolis Santa Catarina Brazil; ^3^ Center for Biotechnology, Institute of Informatics Federal University of Rio Grande do Sul (UFRGS) Porto Alegre Brazil; ^4^ Department of Biochemistry, Center for Biological Sciences Federal University of Santa Catarina Florianópolis Brazil

**Keywords:** bioactive compounds, chlorpyrifos, enzymatic modulation, neuroprotection, organophosphate

## Abstract

Chlorpyrifos (CP) is an organophosphate insecticide that induces neurotoxicity through acetylcholinesterase (AChE) inhibition and redox imbalance, raising environmental and occupational health concerns. Plant‐derived compounds from *Senecio brasiliensis*, native to the Pampa biome, offer multitarget therapeutic potential. This study evaluated the protective effects of the hydroalcoholic extract of *S. brasiliensis* (HESB) against CP‐induced toxicity in *Drosophila melanogaster* (Harwich strain). Adult females (3–5 days old) were exposed for 48 h to control (1% sucrose), CP (0.25 ppm), HESB (1 mg/mL) or CP + HESB. CP increased mortality (37.3% ± 6.6%; *p* < 0.001) and impaired locomotion in the negative geotaxis (17.2 ± 3.0 s; *p* < 0.001) and footbridge (52.2 ± 5.2 s) assays, decreased AChE, GST and CAT activity (−31.8%, −45.8% and −72.3%, respectively; *p* = 0.006, 0.025 and 0.006), elevated ROS (+97.0%; *p* = 0.007), nitrite (+13.1%; *p* = 0.006) and TBARS (+25.6%; *p* = 0.010), depleted NPSH (−29.2%; *p* = 0.003) and PSH (−35.9%; *p* = 0.013) and reduced cell viability (−17.9%; *p* = 0.013). HESB co‐treatment attenuated all CP‐induced alterations, bringing parameters to control‐comparable values (*p* > 0.05). These findings indicate that HESB exerts neuroprotective effects against CP toxicity through modulation of cholinergic function, attenuation of oxidative and nitrosative stress and preservation of thiol homeostasis.

## Summary

- Chlorpyrifos induces mortality, locomotor deficits, AChE inhibition, oxidative stress, thiol depletion and cell viability loss in *Drosophila melanogaster* .
- Hydroalcoholic extract of *Senecio brasiliensis* (HESB) reverses all CP‐toxicity parameters.
- HESB restores cholinergic function, redox homeostasis and thiol levels, positioning it as a promising natural neuroprotective agent against pesticide toxicity.

## Plain Language Summary

Chlorpyrifos is a pesticide widely used in agriculture, but it can damage the nervous system of humans and animals. In this study, we tested whether an extract from a Brazilian plant called *Senecio brasiliensis* (‘Maria‐Mole’) could protect against this damage using fruit flies as a model. The pesticide caused death, movement problems, nerve damage and increased harmful oxidative molecules, while depleting the flies' natural defences. However, when flies received the plant extract together with the pesticide, all these harmful effects were reversed. Our results suggest that this plant extract may be a promising natural strategy to protect against pesticide‐induced toxicity.

## Graphical abstract

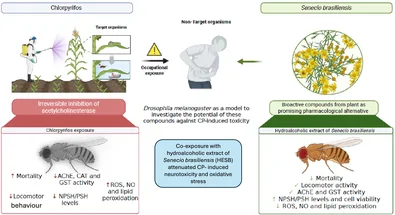

## 1 Introduction

Chlorpyrifos (CP) is one of the most widely used organophosphate pesticides (OPs) globally, and its widespread agricultural application raises significant environmental and public health concerns, particularly regarding its neurotoxic effects in non‐target organisms, including pollinators, aquatic species and humans [1, 2, 3]. The primary toxicological mechanism of CP involves the irreversible inhibition of acetylcholinesterase (AChE), the enzyme responsible for hydrolysing acetylcholine (ACh) at neuronal synapses [4]. AChE inhibition leads to ACh accumulation and sustained cholinergic overstimulation, culminating in neurotoxicity [5]. Beyond cholinergic dysfunction, CP also disrupts cellular redox balance through excessive reactive oxygen species (ROS) generation and depletion of antioxidant defences, compounding neurological damage [6, 7, 8, 9]. Together, these mechanisms highlight the urgent need for effective strategies to counteract CP‐induced toxicity, as recent studies have also identified acute organophosphate poisoning as a potential risk factor for cardiovascular disease [9, 10].

Plant‐derived compounds have emerged as promising candidates in this context, given their multitarget antioxidant and neuroprotective properties [10]. Brazil's rich biodiversity, particularly within the Asteraceae family, represents a valuable source of such bioactive molecules [10, 11]. Within this family, the genus *Senecio*, notably present in southern and southeastern Brazil, has been highlighted for its therapeutic potential, with documented anti‐inflammatory, antioxidant, cytotoxic and neuroprotective activities across several species [12, 13, 14, 15, 16, 17]. Among these species, *Senecio brasiliensis*, native to the South American Pampa biome and popularly known as ‘Maria‐Mole’, is particularly noteworthy for its rich phenolic and flavonoid composition, which underlies its documented anti‐inflammatory and antioxidant properties [12, 18]. The therapeutic use of *S. brasiliensis* , however, warrants caution, as species within the *Senecio* genus are known producers of pyrrolizidine alkaloids (PAs)—hepatotoxic metabolites associated with toxicity in grazing animals, including horses and cattle [19].

Despite this promising pharmacological profile, no study has yet investigated the protective potential of *S. brasiliensis* —or any *Senecio* species—in a model of organophosphate‐induced toxicity, representing a clear and relevant research gap. To address this gap, *Drosophila melanogaster* offers a well‐established platform for assessing neurotoxicity and protective compounds. Conserved cholinergic and redox pathways, combined with genetic tractability, enable rapid in vivo screening [6, 7, 20]. This study therefore evaluated the neuroprotective and antioxidant effects of a hydroalcoholic extract of *S. brasiliensis* (HESB) against CP‐induced toxicity in *D. melanogaster* , hypothesizing that HESB confers protection primarily through its antioxidant properties.

## 2 Materials and Methods

The study was conducted in accordance with the Basic & Clinical Pharmacology & Toxicology policy for experimental and clinical studies [21]. The study was also conducted in accordance with the Basic & Clinical Pharmacology & Toxicology policy for natural products [22].

### 2.1 Materials

Sucrose (S5016), reduced glutathione (GSH, G4251), tetramethylethylenediamine (TEMED, T9281), quercetin (Q4951), 5,5‐dithiobis(2‐nitrobenzoic acid) (DTNB, D8130), 1‐chloro‐2,4‐dinitrobenzene (CDNB, 237329), 3‐(4,5‐dimethylthiazol‐2‐yl)‐2,5‐diphenyltetrazolium bromide (MTT, M2128), CP, 2′,7′‐dichlorofluorescein diacetate (DCF‐DA, 35845), resazurin sodium salt (R7017), d‐mannitol (M9647), K2HPO4 (P3786), KH_2_PO_4_ (P0662), 4‐(2‐hydroxyethyl)‐1‐piperazineethanesulfonic acid (HEPES) minimum 99.5% (titration, H3375), albumin from bovine serum (BSA, A6003), Triton X‐100 (T8532) and β‐mercaptoethanol (M6250) were purchased from Sigma‐Aldrich (St. Louis, MO, USA) at the highest analytical grade. All other chemicals and reagents were of the highest analytical grade.

### 2.2 Plant Material and Extract Preparation

*S. brasiliensis* , popularly known as ‘Maria‐Mole’, is a plant native to the South American Pampa biome and recognized for its rich phenolic and flavonoid composition. To obtain a phytochemically characterized extract for use in this study, leaves of *S. brasiliensis* were collected in November 2014 in the municipality of São Gabriel, Rio Grande do Sul, Brazil (30°19′47.5″ S, 54°21′49.7″ W). Botanical identification was performed at the Herbarium of the Federal University of Pampa (UNIPAMPA), where a voucher specimen was deposited (HBEI 1437).

The extract preparation, including the maceration period, was performed according to the methodology described by Matos (1997) [23]. Fresh leaves (526 g) were washed under running water and dried at room temperature, protected from light, for 2 weeks. The dried material was then macerated (crushed/ground) and placed in a hydroalcoholic solution consisting of ethanol (99.9%) and water (1:1, v/v), where it remained for 7 days in a sealed amber bottle protected from light to extract the phytochemical components. The use of a hydroalcoholic solvent facilitates the extraction of a broad range of polar phytochemicals, including flavonoids and phenolic compounds. After maceration, the mixture was filtered through filter paper, and the solvent was concentrated under reduced pressure using a Heidolph rotary evaporator (50°C, approximately 760 mmHg) for about 1 h. Subsequently, the extract was lyophilized to remove residual water, yielding 22 g of crude extract from *S. brasiliensis* leaves (HESB) [23]. The extract was stored at −20°C until use, protected from light and moisture, in accordance with standard recommendations for long‐term preservation of bioactivity in lyophilized biological/phytochemical material [24, 25]. In addition to improving extract stability, the lyophilization process allowed accurate determination of the extract yield.

The chemical composition of HESB has been previously characterized by our group using HPLC‐DAD analysis [18], which identified and quantified the major phenolic constituents: caffeic acid (3.18 mg/g), gallic acid (2.57 mg/g), vitexin (1.65 mg/g), chlorogenic acid (1.63 mg/g), rosmarinic acid (1.40 mg/g) and quercetin (0.42 mg/g). Furthermore, the extract was analysed prior to the experiments, and its biological activity was preserved.

### 2.3 *D. melanogaster* Maintenance

*D. melanogaster* (Harwich lineage) was obtained from our own breeding and maintained under controlled conditions. All experiments were performed with the same strain. Flies were reared in 50‐mm × 85‐mm glass tubes containing 10 mL of standard medium at constant temperature and humidity (25°C ± 1°C; 60%, respectively). The diet consisted of a mixture of coarse medium, corn flour, wheat germ, sugar, powdered milk, salt, soybean flour, rye flour, methylparaben as an antifungal agent (0.08% v/w nipagin) and lyophilized yeast, following the protocol used in our previous studies [18, 26, 27, 28].

### 2.4 Experimental Design

Adult female *D. melanogaster* (3–5 days old) were temporarily immobilized on ice [29] to allow sexing and random distribution into four experimental groups: (i) control (CTL; 1% sucrose), (ii) CP (0.25 ppm in 1% sucrose), (iii) *S. brasiliensis* extract (HESB; 1 mg/mL in 1% sucrose) and (iv) co‐exposure (CP + HESB; both diluted in 1% sucrose). Flies were housed in glass tubes (50 mm × 85 mm) and exposed for 48 h to their respective treatment solutions, which were applied to filter paper (45 mm diameter) placed at the bottom of each tube.

**FIGURE 1:**
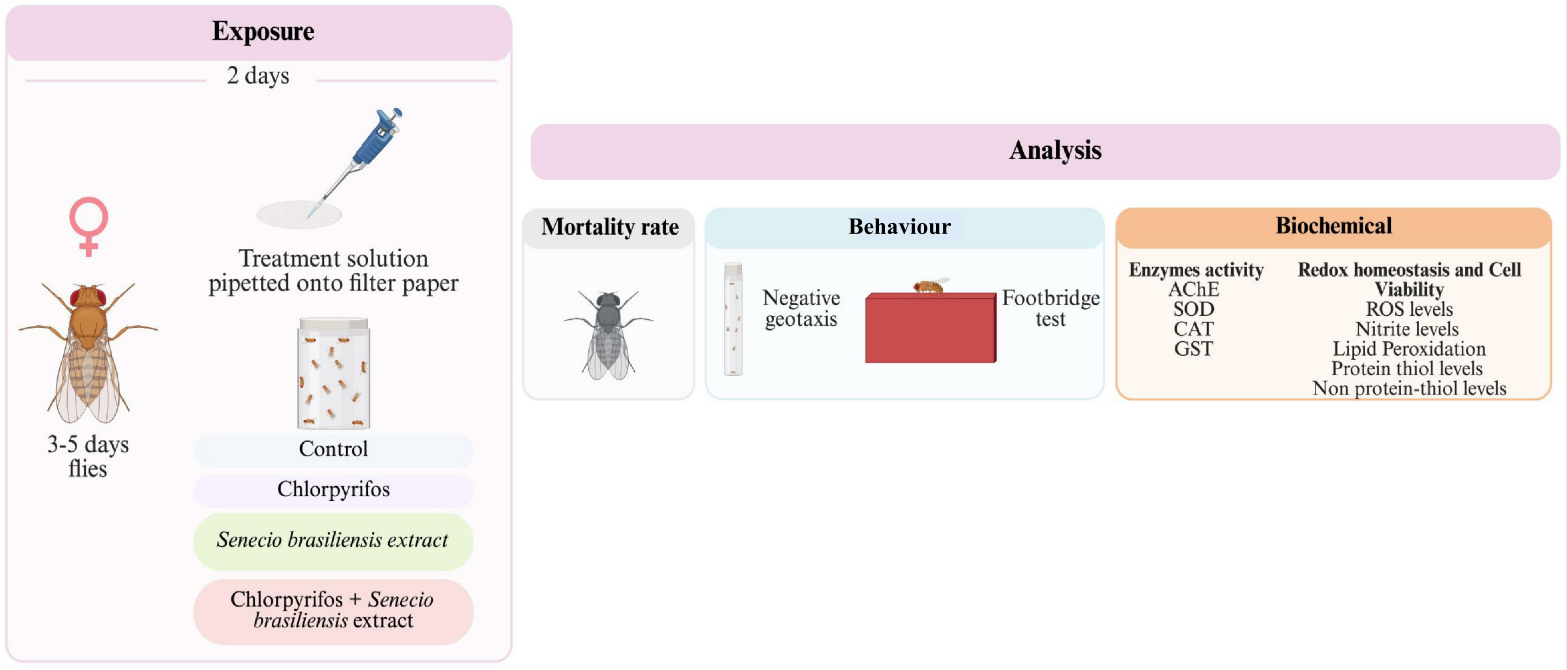
Experimental design. Adult female *Drosophila melanogaster* (3–5 days old) were exposed for 48 h to the respective treatment solutions: (i) control (1% sucrose), (ii) chlorpyrifos (0.25 ppm in 1% sucrose), (iii) *Senecio brasiliensis* extract (HESB; 1 mg/mL in 1% sucrose) and (iv) co‐exposure (CP + HESB; both diluted in 1% sucrose). Flies were randomly distributed into four groups (30 flies per tube; three independent experiments). After exposure, mortality was recorded, followed by behavioural and biochemical assessments.

Following the 48‐h exposure, mortality was recorded for all groups, after which flies were subjected to behavioural and biochemical assessments (Figure 1). For mortality and behavioural assays, the experimental unit consisted of a tube containing 30 flies, with three independent biological replicates per group (total *n* = 90 flies per group). For biochemical assays, pools of 20 flies were homogenized for analysis, with three to five independent replicates per group depending on the assay. For all procedures following exposure, flies were immobilized on ice prior to transfer to test apparatus or homogenization tubes (Figure 1).

The sample sizes employed in this study reflect a standardized protocol consistently used in previous studies from our research group employing the same experimental model [6, 18, 20, 26, 28]. Groups of 30 flies per replicate, with three independent biological replicates (total *n* = 90 flies per group), have been consistently applied across these studies to assess mortality and locomotor performance in *D. melanogaster* exposed to various toxicants, whereas pools of 20 flies per replicate, with three to five independent replicates per group, have been similarly adopted for biochemical assays, reliably yielding sufficient protein concentrations for enzymatic and oxidative stress analyses. No inclusion or exclusion criteria were established a priori; all flies that survived the 48‐h exposure period were included in the analyses, and no data points were excluded. To minimize potential confounders, all assays were performed blind—samples were coded and the researcher conducting the measurements remained unaware of treatment allocation until after data collection. Additionally, all procedures were conducted at the same time of day, and flies from different groups were handled in alternating order to avoid systematic bias.

### 2.5 Mortality Assessment

Mortality was recorded after the 48‐h exposure period. Flies were considered dead when no movement was observed after gentle tapping of the tube. Mortality was quantified as the number of dead flies per sample (replicate), where each sample consisted of 30 flies.

### 2.6 Locomotor Analysis

#### 2.6.1 Negative Geotaxis

The climbing ability of flies was assessed using a negative geotaxis [24]. Groups of 10 flies were transferred to vertical glass tubes (25 cm height × 1.5 cm diameter) closed with the respective lids. After a 30‐min recovery period at 25°C, each tube was gently tapped to bring all flies to the bottom, and the number of flies that climbed at least 6 cm within 10 s was recorded. This procedure was repeated four times consecutively at 20‐s intervals using the same group of flies. Three independent biological replicates were performed for each experimental condition, each with a different group of 10 flies (total *n* = 30 flies per condition).

#### 2.6.2 Footbridge Test

The footbridge test was conducted as described by Gomes et al. [26], with the apparatus design optimized based on previous studies [30]. The set‐up consisted of a narrow horizontal walkway measuring 13 cm in length and 0.5 cm in width. Following the exposure periods, the flies had their wings clipped 3 h prior to testing to prevent flight during the assay. Each fly was placed at the starting point of the walkway and allowed up to 60 s to complete the crossing. The time taken by the flies to complete the path was recorded, and the results were expressed in seconds, with a total of 10 flies per group.

### 2.7 Sample Preparation for Biochemical Analyses

For biochemical assays, except for non‐protein thiol (NPSH) and protein thiol (PSH) assays, a pool of 20 flies per group was homogenized in 1 mL of 20‐mM HEPES buffer (pH 7.0) using a manual homogenizer [31]. The homogenates were subjected to sequential centrifugation steps to separate the supernatant for each assay.

### 2.8 Enzymatic Analyses

For AChE activity, an aliquot of the homogenate was centrifuged at 1000 × g for 5 min at 4°C, and the supernatant was used to measure AChE activity at 412 nm, using ATCh as the substrate [32] (*n* = 4 pools of 20 flies each, total *n* = 80 flies per group).

For the analysis of antioxidant enzymes, the remaining homogenate was centrifuged at 20 000 × g for 30 min at 4°C. The supernatant was then used to determine the activities of catalase (CAT), superoxide dismutase (SOD) and glutathione S‐transferase (GST) [28].

CAT activity was measured by monitoring the clearance of H_2_O_2_ at 240 nm using a reaction mixture composed of 0.05‐M phosphate buffer (pH 7.0), 0.5‐mM EDTA, 10‐mM H_2_O_2_ and 0.012% Triton X‐100 [33] (*n* = 3 pools of 20 flies each, total *n* = 60 flies per group). SOD activity was determined based on the SOD‐mediated inhibition of quercetin autoxidation, which generates superoxide radicals in the presence of *N*,*N*,*N*′,*N*′‐tetramethylethane‐1,2‐diamine (TEMED), with absorbance measured at 405 nm [34] (*n* = 3 pools of 20 flies each, total *n* = 60 flies per group). GST activity was assessed by measuring the conjugation of CDNB with GSH, with absorbance measured at 340 nm [35] (*n* = 4 pools of 20 flies each, total *n* = 80 flies per group).

All assays were performed at room temperature (~25°C) using an Agilent Cary 60 UV/VIS spectrophotometer (Santa Clara, CA), and enzyme activity was expressed as mU/min/mg of protein and as a percentage relative to the control group.

### 2.9 Determination of Cell Viability and ROS Generation

The homogenates were centrifuged at 1000 × g for 10 min at 4°C, and the resulting supernatant was used to measure steady‐state ROS levels through the oxidation of the fluorescent dye 2′,7′‐dichlorofluorescein diacetate (DCFH_2_‐DA) [36] as previously adapted [37]. Fluorescence was recorded at 485 nm (excitation) and 530 nm (emission). Cell viability was assessed using the resazurin assay [38], which quantifies the ability of viable cells to reduce resazurin to the fluorescent product resorufin. Fluorescence of the resorufin product was measured at 544 nm (excitation) and 590 nm (emission). All assays were performed using an EnsPire multimode plate reader (PerkinElmer, Waltham, MA). The results were normalized to total protein content and expressed as a percentage relative to the control group (*n* = 3 pools of 20 flies each, total *n* = 60 flies per group).

### 2.10 Nitric Oxide (NO) Content

NO levels were assessed by measuring nitrite formation using the Griess reagent [39]. The homogenate was centrifuged at 10 000 × g for 10 min at 4°C, and the supernatant was incubated with the Griess reagent (sulfanilamide 1% in 5% phosphoric acid with NED 0.1% in water) [27]. Absorbance was measured at 535 nm, and nitrite concentration was determined from a standard curve using an EnsPire multimode plate reader (PerkinElmer, Waltham, MA). The results were normalized to total protein content and expressed as a percentage relative to the control group (*n* = 3 pools of 20 flies each, total *n* = 60 flies per group).

### 2.11 Lipid Peroxidation

Lipid peroxidation was assessed using the thiobarbituric acid‐reactive substances (TBARS) method without prior homogenization [26, 40]. Groups of 20 whole flies were placed in glass tubes containing TBA (0.005% in 20% TCA) and incubated at 95°C for 30 min (*n* = 5 replicates of 20 flies each, total *n* = 100 flies per group). TBA reacts with aldehydic byproducts of lipid oxidation, forming a coloured complex detected at 532 nm in a microplate reader. Results were expressed as absorbance relative to the control.

### 2.12 Thiol Levels

The thiol content, including NPSH and PSH levels, was measured using the method described by Ellman (1959) [32] with modifications [37]. Briefly, 20 flies were homogenized in 1 mL of 0.5‐M Tris/HCl (pH 8.0) at 4°C. The homogenate was separated for protein quantification, and 0.5‐M perchloric acid was added. The samples were then centrifuged at 9500 × g for 5 min at 4°C, and the supernatant was incubated with DTNB in a 96‐well plate. NPSH levels were quantified by measuring absorbance at 412 nm.

For PSH analysis, the pellet was resuspended in the same homogenization solution and incubated with DTNB. Absorbance was recorded at 412 nm, with a second reading at 650 nm to subtract turbidity. All assays were performed using an EnsPire multimode plate reader (PerkinElmer, Waltham, MA). The results were normalized to total protein content and expressed as μmol SH/mg protein as a percentage relative to the control group (*n* = 4 pools of 20 flies each, total *n* = 80 flies per group).

### 2.13 Protein Quantification

Protein concentration in the samples was determined using the Bradford method [41], which relies on the interaction between Coomassie Brilliant Blue G‐250 dye and protein molecules, resulting in a colorimetric shift measurable by spectrophotometry.

### 2.14 Statistical Analysis

Normality was assessed using the Shapiro–Wilk test. Parametric data are expressed as mean ± SEM and were analysed by one‐way ANOVA followed by Tukey's post hoc test or unpaired Student's *t*‐test. Nonparametric data are expressed as median ± interquartile range (IQR) and were analysed by Kruskal–Wallis followed by Dunn's post hoc test. Significance was set at *p* ≤ 0.05.

## 3 Results

### 3.1 HESB Co‐Exposure Protects Against CP‐Induced Mortality and Locomotor Impairments

**FIGURE 2:**
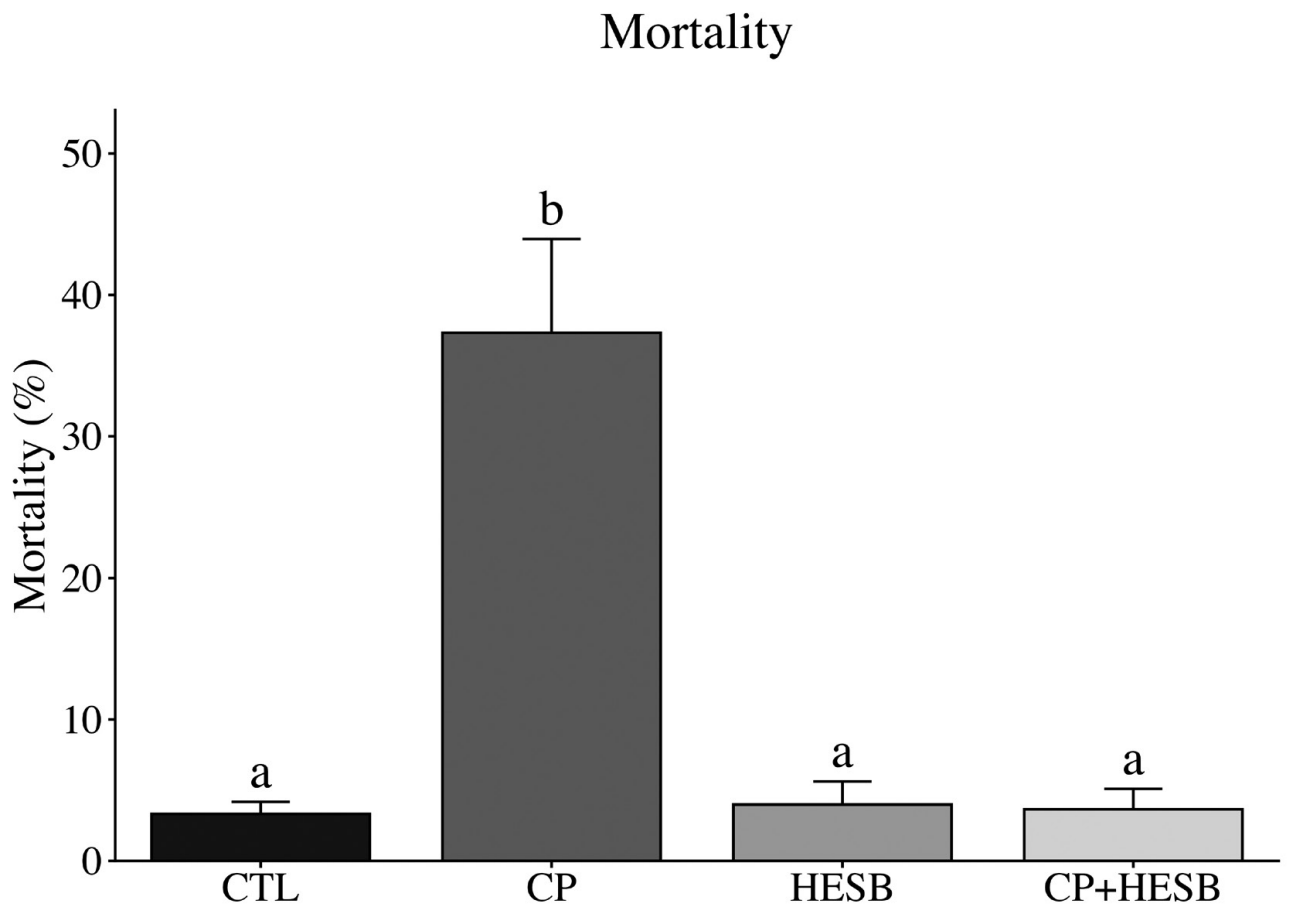
Effects of HESB on CP‐induced mortality in *Drosophila melanogaster* . CP exposure significantly increased mortality (37.3% ± 6.6%) compared to controls (3.3% ± 0.9%; *p* < 0.001), whereas co‐treatment with HESB (3.7% ± 1.4%) prevented this effect. HESB alone did not alter survival (4.0% ± 1.6%). Data represent mean ± SEM (*n* = 10 tubes per group, 30 flies per tube). One‐way ANOVA followed by Tukey's post hoc test was used for analysis. Different letters indicate significant differences between groups (a, b, c) (*p* < 0.05).

CP exposure significantly increased mortality to 37.3% ± 6.6% compared with 3.3% ± 0.9% in controls (*p* < 0.001; Figure 2). Co‐treatment with HESB (3.7% ± 1.4%) and HESB alone (4.0% ± 1.6%) did not differ from controls.

**FIGURE 3:**
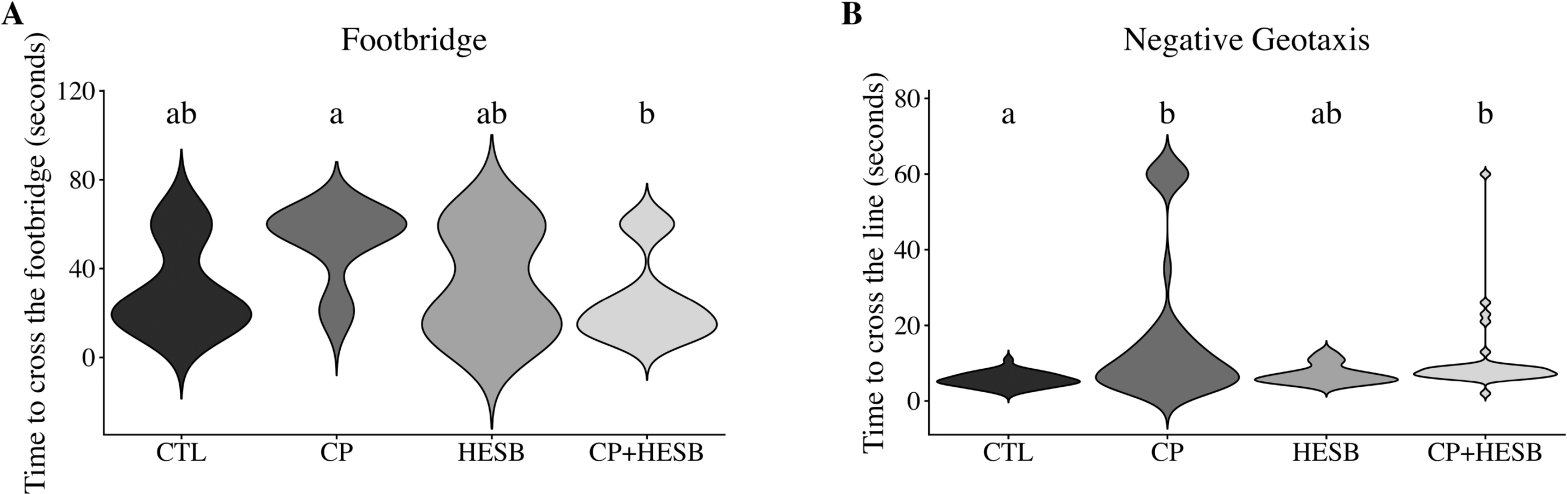
HESB attenuates CP‐induced motor deficits in *Drosophila melanogaster*. (A) In the footbridge test, CP‐exposed flies showed delayed task completion (52.2 ± 5.2 s) compared to controls (31.5 ± 6.2 s). (B) In the negative geotaxis assay, CP‐exposed flies took longer to reach the 6‐cm mark (17.2 ± 3.0 s) than controls (5.6 ± 0.3 s; *p* < 0.001). Co‐treatment with HESB attenuated locomotor impairments in both tests. Data are expressed as mean ± SEM (footbridge: *n* = 10 flies per group; negative geotaxis: *n* = 30 flies per group). Kruskal–Wallis followed by Dunn's post hoc test (Bonferroni correction). Different letters indicate significant differences between groups (a, b, c) (*p* < 0.05).

Locomotor behaviour was negatively affected by CP, as demonstrated in the footbridge assay (Figure 3A), where CP‐exposed flies showed delayed task completion (52.2 ± 5.2 s) compared with controls (31.5 ± 6.2 s; *p* = 0.296). Although this comparison did not reach statistical significance, the biological relevance of the CP effect is supported by the CP group mean reaching the maximum recordable time of 60 s and by the clear and significant recovery observed in co‐treated flies, which completed the crossing in 25.4 ± 6.1 s—significantly lower than CP (*p* = 0.040) and not significantly different from controls (*p* = 1.000).

In the negative geotaxis assay (Figure 3B), CP significantly increased crossing time (17.2 ± 3.0 s) relative to controls (5.6 ± 0.3 s; *p* < 0.001). CP + HESB flies completed the task in 10.1 ± 1.5 s (179.9% ± 26.2% of control; *p* = 0.156 vs. CTL), representing a 41% reduction relative to CP (306.2% ± 54.2% of control), though this did not reach statistical significance (*p* = 1.000 vs. CP), partly due to a single individual requiring the maximum recording time of 60 s.

### 3.2 HESB Co‐Exposure Modulates AChE Activity and Antioxidant Enzymes

**FIGURE 4:**
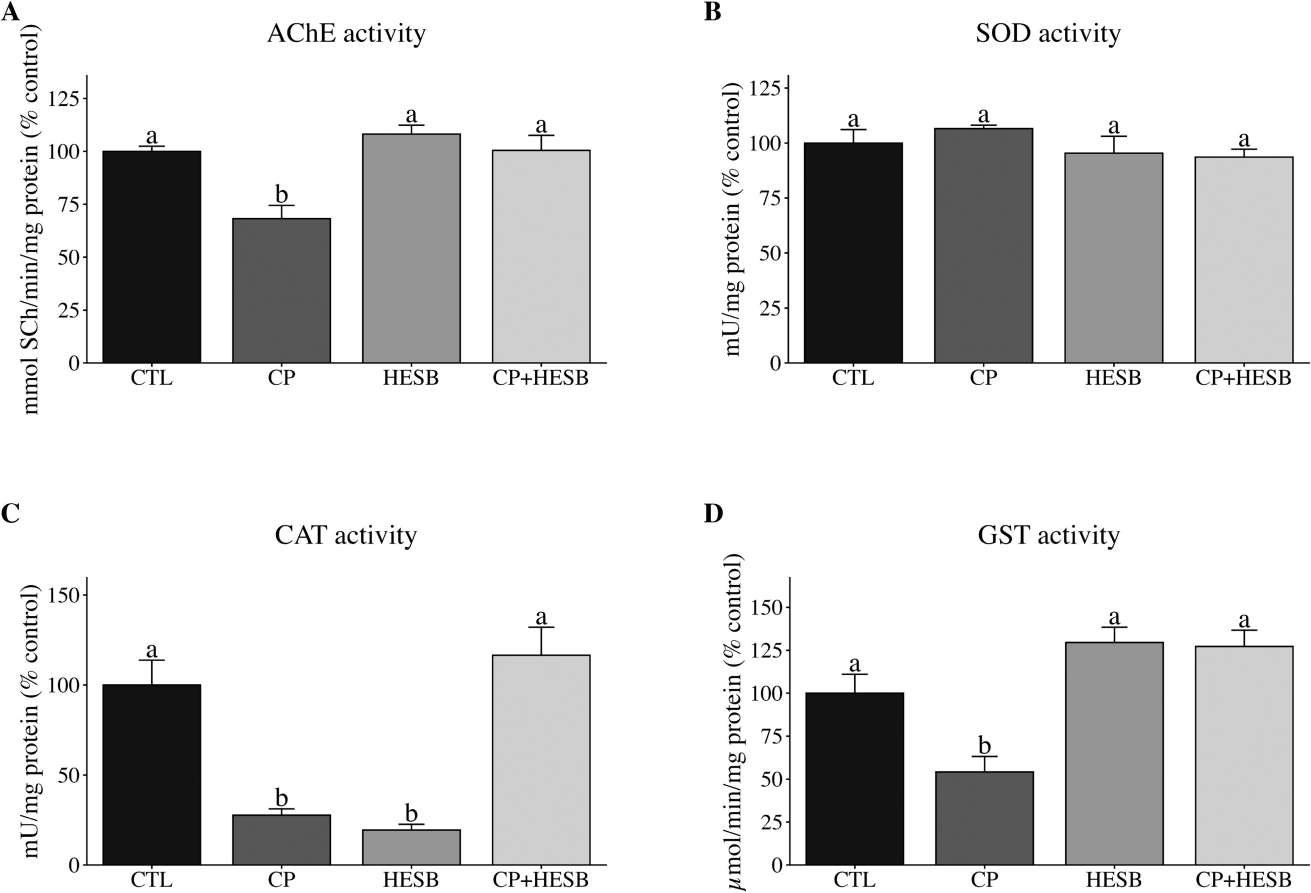
Modulation of cholinergic and antioxidant enzymes by HESB in CP‐exposed *Drosophila melanogaster* . (A) AChE activity was significantly reduced by CP (68.2% ± 6.2%; *p* = 0.006 vs. CTL) and attenuated by HESB co‐exposure (100.5% ± 7.1%; *p* = 0.005 vs. CP). (B) SOD activity showed no significant differences among groups (*p* > 0.05). (C) CAT activity was suppressed by CP (27.7% ± 3.5%; *p* = 0.006) and HESB alone (19.4% ± 3.2%; *p* = 0.003), whereas co‐exposure modulated CAT to levels not significantly different from controls (116.5% ± 15.7%; *p* = 0.704). (D) GST was reduced by CP (54.2% ± 9.0%; *p* = 0.025) and modulated by HESB co‐exposure (127.3% ± 9.5%; *p* < 0.001 vs. CP). Enzyme activities are expressed as a percentage of control values, with AChE activity measured in SCH/min/mg protein, CAT and SOD activities in mU/mg protein and GST activity in μmol/min/mg protein. Parametric data are expressed as a percentage relative to the control group and presented as mean ± SEM. For AChE and GST: *n* = 4 pools of 20 flies each, total *n* = 80 flies per group. For CAT and SOD: *n* = 3 pools of 20 flies each, total *n* = 60 flies per group. One‐way ANOVA followed by Tukey's post hoc test was used for analysis. Different letters indicate significant differences between groups (a, b, c) (*p* < 0.05).

CP exposure significantly inhibited AChE activity (68.2% ± 6.2% vs. 100.0% ± 2.5% in controls; *p* = 0.006), an effect attenuated by HESB co‐exposure (100.5% ± 7.1%; *p* = 0.005 vs. CP; Figure 4A). CAT activity was suppressed by both CP (27.7% ± 3.5%; *p* = 0.006) and HESB alone (19.4% ± 3.2%; *p* = 0.003), but co‐exposure restored it above control levels (116.5% ± 15.7%; *p* = 0.704 vs. CTL; Figure 4C). SOD activity remained unchanged across all groups (*p* > 0.05; Figure 4B). GST was significantly reduced by CP (54.2% ± 9.0%; *p* = 0.025 vs. CTL) but restored by HESB co‐exposure (127.3% ± 9.5%; *p* < 0.001 vs. CP; Figure 4D).

### 3.3 HESB Protects Against CP‐Induced Oxidative Damage and Improves Thiol Balance and Cell Viability

**FIGURE 5:**
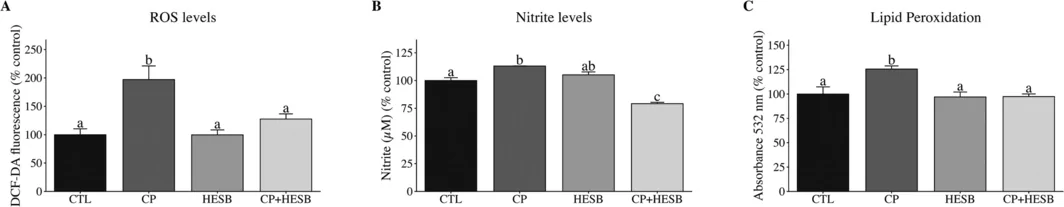
HESB attenuates oxidative stress induced by CP in *Drosophila melanogaster* . CP elevated (A) ROS levels (197.0% ± 24.1%; *p* = 0.007), (B) nitrite content (113.1% ± 0.1%; *p* = 0.006) and (C) lipid peroxidation (TBARS; 125.6% ± 3.2%; *p* = 0.010) compared to controls. HESB co‐exposure attenuated all parameters to values comparable to controls (ROS: 127.5% ± 9.3%; nitrite: 79.1% ± 1.2%; TBARS: 97.4% ± 2.7%; all *p* > 0.05 vs. CTL). Parametric data are expressed as a percentage relative to the control group and presented as mean ± SEM. For ROS and nitrite: *n* = 3 pools of 20 flies each, total *n* = 60 flies per group. For TBARS: *n* = 5 replicates of 20 flies each, total *n* = 100 flies per group. One‐way ANOVA followed by Tukey's post hoc test was used for analysis. Different letters indicate significant differences between groups (a, b, c) (*p* < 0.05).

**FIGURE 6:**
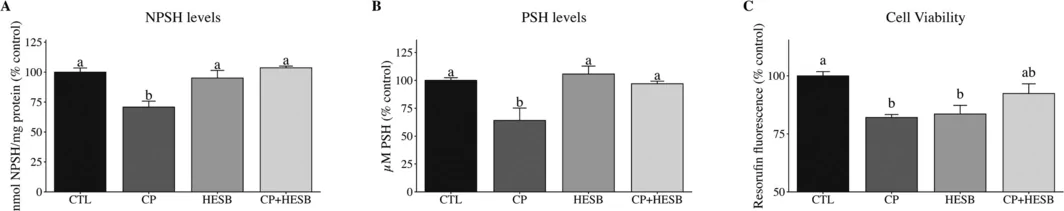
HESB modulates thiol status and cell viability in *Drosophila melanogaster* exposed to CP. (A) CP reduced NPSH levels (70.8% ± 5.0%; *p* = 0.003 vs. CTL), an effect attenuated by HESB co‐exposure (103.6% ± 1.5%; *p* = 0.001 vs. CP). (B) PSH content was similarly reduced by CP (64.1% ± 11.2%; *p* = 0.013) and modulated by co‐exposure (97.0% ± 2.3%; *p* = 0.023 vs. CP). (C) Cell viability was reduced by CP (82.1% ± 1.3%; *p* = 0.013) and HESB alone (83.6% ± 3.7%; *p* = 0.020), whereas co‐exposure preserved viability at values comparable to controls (92.4% ± 4.2%; *p* = 0.346 vs. CTL). For NPSH and PSH: *n* = 4 pools of 20 flies each, total *n* = 80 flies per group. For resazurin: *n* = 3 pools of 20 flies each, total *n* = 60 flies per group. Parametric data are expressed as a percentage relative to the control group and presented as mean ± SEM. One‐way ANOVA followed by Tukey's post hoc test was used for analysis. Different letters indicate significant differences between groups (a, b, c) (*p* < 0.05).

HESB co‐exposure attenuated ROS generation (CP: 197.0% ± 24.1%; CP + HESB: 127.5% ± 9.3%; *p* = 0.040 vs. CP; Figure 5A), decreased lipid peroxidation (CP: 125.6% ± 3.2%; CP + HESB: 97.4% ± 2.7%; *p* = 0.005 vs. CP; Figure 5C) and prevented excessive nitrite accumulation (CP: 113.1% ± 0.1%; CP + HESB: 79.1% ± 1.2%; *p* < 0.001 vs. CP; Figure 5B). CP also depleted NPSH (70.8% ± 5.0%; *p* = 0.003 vs. CTL) and PSH (64.1% ± 11.2%; *p* = 0.013 vs. CTL), effects reversed by HESB co‐treatment (NPSH: 103.6% ± 1.5%; *p* = 0.001 vs. CP; PSH: 97.0% ± 2.3%; *p* = 0.023 vs. CP; Figure 6A,B). Cellular metabolic activity was significantly impaired by CP (82.1% ± 1.3%; *p* = 0.013 vs. CTL). HESB alone also reduced viability (83.6% ± 3.7%; *p* = 0.020 vs. CTL), similarly to CP. Notably, co‐exposure preserved viability at values not significantly different from controls (92.4% ± 4.2%; *p* = 0.346 vs. CTL; Figure 6C).

## 4 Discussion

Brazil ranks among the world's largest consumers of pesticides, a status driven by its intensive agricultural practices and vast territorial extension [42]. CP, one of the most widely used active ingredients, ranked fifth in national sales in 2022, with approximately 7160 tons sold [43]. CP's toxicity stems from irreversible AChE inhibition, triggering neurotoxic effects and raising concerns about protective strategies [1, 5]. Recently, plant‐derived compounds have emerged as promising agents to counteract CP‐induced toxicity due to their antioxidant and anti‐inflammatory properties [6, 20]. Building on this potential, our study investigated the neuroprotective effects of the HESB in *D. melanogaster* .

HESB reduced CP‐induced mortality and locomotor deficits, with co‐treated flies showing markedly improved survival compared to those exposed to CP alone, consistent with our group's previous observations under the same experimental conditions [6]. Although direct evidence of *S. brasiliensis* protective effects against pesticide toxicity was limited prior to this study, its phytochemical profile has been previously characterized by our group [18]. HESB is rich in phenolic acids and flavonoids, including gallic acid (2.57 mg/g), caffeic acid (3.18 mg/g), vitexin (1.65 mg/g), chlorogenic acid (1.63 mg/g), rosmarinic acid (1.40 mg/g) and quercetin (0.42 mg/g). This phytochemical diversity, characterized by well‐documented antioxidant, cytoprotective and neuroprotective properties [6, 18], provides a compelling mechanistic basis for the broad protective effects observed across multiple endpoints in the present study.

Locomotor impairments—hallmark signs of CP poisoning linked to AChE inhibition—were attenuated by HESB co‐treatment, as evidenced by improved performance in negative geotaxis and footbridge assays. The negative geotaxis assay measures climbing ability against gravity, a reliable indicator of neuromotor function, whereas the footbridge test evaluates coordinated horizontal movement and balance, providing additional insights into motor function [6, 28, 44, 45]. HESB‐treated flies showed faster climbing responses and reduced crossing times, reflecting improved motor capacity. In the footbridge assay, HESB co‐exposure produced a statistically significant reduction in crossing time relative to CP, with co‐treated flies performing comparably to controls. In the negative geotaxis assay, co‐treated flies showed a 41% reduction in mean crossing time relative to CP, a biologically meaningful attenuation that did not reach full statistical separation from CP, largely driven by a single individual in the CP + HESB group that required the maximum recording time—a value that, while not excludable as an outlier, disproportionately elevated the group mean relative to the median (8.0 vs. 8.5 s in CP).

These behavioural improvements were accompanied by higher AChE activity in co‐treated flies, pointing to a modulatory effect of HESB on cholinergic function. The maintenance of GST activity at control levels in co‐treated flies—significantly reduced by CP alone—supports the hypothesis that HESB preserved the flies' intrinsic detoxification capacity, thereby limiting CP bioavailability and its inhibitory action on AChE, given the well‐established role of GST enzymes in organophosphate conjugation and elimination [35]. Corroborating this interpretation, polyphenolic constituents of HESB may also modulate cytochrome P450‐dependent bioactivation of CP to its neurotoxic oxon metabolite, further reducing the availability of the AChE‐inhibiting species—a mechanism previously reported for plant‐derived polyphenols against CP toxicity [46, 47].

Across locomotor performance and AChE activity, these findings support a neuroprotective role of HESB, consistent with the growing recognition of the therapeutic potential of *Senecio* species in neurological contexts [12, 14, 16, 17]. Phenolic acids and flavonoids present in HESB are known to modulate key neuronal survival pathways, including AKT and ERK signalling [17], and to broadly counteract neurotoxicity across different experimental models [48, 49], reinforcing the phytochemical basis for the improvements observed in the present study.

Beyond cholinergic dysfunction, CP disrupts cellular redox balance through excessive ROS generation and depletion of antioxidant defences [6]. The suppression of CAT alongside unchanged SOD activity suggests H_2_O_2_ accumulation, which—combined with mitochondrial superoxide generation and its subsequent reaction with NO to form peroxynitrite [50, 51]—propagates a broad spectrum of oxidative and nitrosative damage, as evidenced by elevated nitrite levels, increased lipid peroxidation and depletion of both non‐protein and protein thiol contents in CP‐exposed flies. Peroxynitrite is not only a potent oxidant but also a key mediator of inflammatory signalling [50, 51], and its attenuation by HESB co‐treatment may therefore reflect a broader anti‐inflammatory dimension consistent with the previously documented anti‐inflammatory properties of *S. brasiliensis* [14].

Of particular interest was the observation that HESB alone also reduced CAT activity, a finding that is mechanistically contextualized by the well‐documented capacity of flavonoids to modulate antioxidant enzyme activity depending on the prevailing redox environment. Under basal redox conditions, flavonoids can interact directly with the haem centre of CAT through hydrogen bonding, promoting the conversion of the active Compound I intermediate to the unreactive Compound II form, whose accumulation progressively inhibits enzyme activity [52, 53]. In the absence of significant oxidative challenge, insufficient H_2_O_2_ is available to outcompete this inhibitory interaction, and the net result is reduced CAT activity. This mechanism was formally characterized by Krych and Gebicka (2013) [53], who demonstrated in vitro that multiple flavonoids—including myricetin, epicatechin gallate and epigallocatechin gallate—inhibit CAT through Compound II formation, with inhibitory potency related to the molecular structure of the flavonoid. Krych et al. (2014) [52] further established that hydrogen bonding between flavonoids and the enzyme stabilizes this inactive state. Notably, quercetin—one of the flavonoids identified in HESB [18]—has been specifically shown to inhibit CAT through conformational changes induced via non‐competitive binding interactions [54, 55], providing a direct phytochemical link between HESB composition and the observed enzyme response.

Under CP‐induced oxidative stress, however, the radical scavenging properties of flavonoids become predominant, with these compounds intercepting reactive species before they can compromise CAT catalytic function—a context‐dependent dual behaviour further supported by Speisky et al. (2022) [56], who demonstrated that flavonoid oxidation under oxidative challenge generates electrophilic metabolites capable of activating the Nrf2–Keap1 pathway and amplifying endogenous antioxidant defences.

Co‐treatment with HESB mitigated the oxidative disturbances induced by CP, with CAT activity maintained at levels comparable to control, accompanied by significant attenuation of ROS generation, nitrite accumulation, lipid peroxidation and thiol depletion. These findings collectively indicate that HESB modulates CP‐induced redox imbalance through a multitarget mechanism encompassing both enzymatic and non‐enzymatic antioxidant defences. The preservation of thiol content reflects the capacity of phenolic acids and flavonoids present in HESB to sustain GSH availability through enhanced synthesis via glutamate‐cysteine ligase and glutathione synthetase [57], replenishing antioxidant reserves consumed under oxidative stress.

Cellular metabolic activity, assessed by the resazurin reduction assay [38], was significantly reduced by CP exposure alongside overt oxidative damage and increased mortality, consistent with organophosphate‐induced mitochondrial dysfunction [6, 20]. Although HESB alone also reduced resorufin fluorescence to levels comparable to CP‐exposed flies, this effect reflects a direct modulation of mitochondrial reductive capacity rather than cytotoxicity, given the absence of redox disturbances or increased mortality in this group. Polyphenols and flavonoids are recognized to exert direct effects on mitochondrial function through mechanisms independent of ROS scavenging, including modulation of electron transport chain complexes, mitochondrial transmembrane potential and oxidative phosphorylation coupling [58], which may alter mitochondrial reductive capacity without compromising cellular integrity. Under co‐exposure conditions, HESB attenuated the CP‐induced impairment of cellular metabolic activity, with co‐treated flies presenting intermediate values not significantly different from control.

The broad protective profile described here corroborates the pharmacological potential increasingly attributed to *Senecio* species. The in vitro radical scavenging activity of HESB, previously characterized by our group through DPPH and ABTS assays [18], is consistent with findings reported across the genus: *Senecio jacobaea* [59], *Senecio clivicolus* [16], *Senecio glaucus* [60] and *Senecio nutans* [61] all exhibit significant free radical neutralization capacity, and comparative studies evaluating multiple species simultaneously have demonstrated that phenolic richness and redox‐modulating properties are broadly distributed features within *Seneco*.These properties extend beyond direct radical scavenging—*Senecio graciliflorus* protects human neuroblastoma cells through AKT and ERK neuronal survival pathways [18], and *Senecio serratuloides* attenuates oxidative stress and cardiovascular dysfunction in rodents [54]—collectively suggesting that the genus operates through conserved cytoprotective mechanisms.

Together with our findings, these data—spanning cell‐free assays, human‐derived neuronal cells and rodent models—reinforce the pharmacological potential of *Senecio* species and support their further exploration in vertebrate systems. Future investigations would benefit from examining the effects of individual phytochemical constituents, distinct exposure protocols and chronic toxicity scenarios. Given the significant public health burden associated with CP exposure in agricultural and occupational settings, such studies—including evaluation of sex‐dependent differences in response—would substantially advance the translational potential of these findings.

## 5 Conclusion

The present study demonstrates, for the first time, that HESB exerts neuroprotective effects against CP‐induced toxicity in *D. melanogaster* , extending the growing body of evidence supporting the pharmacological potential of *Senecio* species. Protection was observed across multiple endpoints, encompassing cholinergic function, antioxidant enzyme activity, thiol homeostasis and cellular metabolic activity. These findings position HESB as a promising candidate for further investigation in the context of pesticide‐induced neurotoxicity and highlight the therapeutic relevance of *S. brasiliensis* against environmentally significant toxicants.

## Author Contributions

**Maria Vitória Takemura Mariano:** writing – review and editing, writing – original draft, methodology, investigation, conceptualization. **Giany Gabriely Padão dos Santos:** methodology, investigation. **Ana Beatriz Guedes de Souza:** methodology, investigation. **Thaís de Camargo Neves:** methodology, investigation. **Franklin Vinny Medina Nunes:** methodology. **Pedro Henrique Vicenzi:** methodology. **Karen Kich Gomes:** methodology, investigation. **Mauro Eugênio Medina Nunes:** methodology, writing – review and editing. **Marcelo Farina:** project administration, funding acquisition. **Jeferson Luis Franco:** supervision, investigation, funding acquisition. **Thaís Posser:** writing – review and editing, supervision, resources, investigation, formal analysis, data curation.

## Funding

This study was supported by Coordenação de Aperfeiçoamento de Pessoal de Nível Superior (CAPES) (001) and Conselho Nacional de Desenvolvimento Científico e Tecnológico (CNPq) (405426/2021‐6, 173307/2023‐0, 406442/2023‐3). JLF and TP are CNPq research fellows (311829/2023‐1 and 307316/2022‐0, respectively), Federal University of Pampa (Support for Research Groups).

## Conflicts of Interest

The authors declare no conflicts of interest.

## Data Availability

Data are available from the corresponding author upon request.
